# Cutting-edge research frontiers in oral cavity vaccines for respiratory diseases: a roadmap for scientific advancement

**DOI:** 10.3389/fcimb.2024.1388222

**Published:** 2024-06-26

**Authors:** Erwan Sallard, Malik Aydin

**Affiliations:** ^1^ Virology and Microbiology, Center for Biomedical Education and Research (ZBAF), School of Medicine, Faculty of Health, Witten/Herdecke University, Witten, Germany; ^2^ Laboratory of Experimental Pediatric Pneumology and Allergology, Center for Biomedical Education and Research, School of Life Sciences (ZBAF), Faculty of Health, Witten/Herdecke University, Witten, Germany; ^3^ Institute for Medical Laboratory Diagnostics, Center for Clinical and Translational Research, Helios University Hospital Wuppertal, Witten/Herdecke University, Wuppertal, Germany

**Keywords:** vaccine, mucosal vaccine, respiratory disease, vaccinology, vector

## Abstract

Intramuscular vaccines present limitations in eliciting robust mucosal immunity and preventing respiratory pathogens transmission. Sublingual vaccine administration offers promising advantages, including interconnected mucosal protection. Despite these advantages, only a few clinical trials have explored sublingual vaccines, underscoring the necessity of optimizing next-generation vaccine formulas. Critical research priorities include understanding vector behavior in the oral environment, understanding their interactions with mucosal immunity and developing formulations enabling sustained mucosal contact to facilitate efficient transduction. Consequently, tonsil organoids, as representative human mucosal models, could offer critical insights into sublingual immunization. Thus, a multi-disciplinary approach integrating pharmacological, immunological, and manufacturing considerations is pivotal for sublingual vaccines in targeting pathogen-aggravated prevalent respiratory diseases including asthma, COPD and lung cancer, as well as the antimicrobial resistance crisis.

## Main

Intramuscular administration remains the paradigm for vaccination due to well-understood systemic immune responses and highly standardized delivery procedures. However, intramuscular vaccines exhibit certain disadvantages including suboptimal induction of mucosal immunity, as stated in the literature. For example, the intramuscular polio vaccines induce lower antibody secretion in the gastrointestinal tract than their enteric counterpart (the Sabin vaccine) (Ogra, 1984). This is believed to be the main reason of their lower efficacy (Onorato et al., 1991; WHO Collaborative Study Group on Oral and Inactivated Poliovirus Vaccines, 1997), as supported by the negative correlation observed between intestinal antibody titers and poliovirus shedding (Swartz et al., 2008). Likewise, preclinical head-to-head comparisons of various immunization routes consistently found that mucosal administration induced superior protection against certain pathogens (Belyakov and Ahlers, 2009) including *M. tuberculosis* (Wang et al., 2004; Forbes et al., 2008), influenza (Perrone et al., 2009) and HIV (Belyakov et al., 2001) compared to intramuscular or intradermal administration. Suboptimal mucosal responses may contribute to the mediocre effectiveness of intramuscular coronavirus disease (COVID) vaccines in preventing pathogen transmission, despite them providing adequate disease prevention due to the systemic response. Starting in 2022, several mucosal COVID vaccines have been approved (Pilapitiya et al., 2023), among which the adenovirus-vectored Convidecia vaccine was shown to induce higher neutralizing antibody titers as an inhaled vaccine than after intramuscular delivery (Li et al., 2022).

Consequently, vaccines should ideally be delivered at the site of infection of the targeted pathogen, aiming at stimulating both systemic immunity and the resident lymphoid tissue of the vaccinated mucosa. Mucosal immunity relies on secretions such as saliva that provide the first barrier to microorganisms, while mucosa-associated lymphoid tissue (MALT) plays a robust defense role (Kiyono and Azegami, 2015). To establish effective protection, antigens need to cross the mucus and epithelial layers, be detected by sentinel cells including dendritic and Langerhans cells, and be reactogenic enough so that these antigen-presenting cells activate MALT cellular and humoral responses, the latter relying crucially on secreted dimeric Immunoglobulin (Ig)A antibodies (Kiyono and Azegami, 2015; Paris et al., 2021).

Secreted IgGs are also known to play a crucial role at least in the lower respiratory and female reproductive tracts (Parr and Parr, 1998; Renegar et al., 2004). Activated CD4^+^ T cells can differentiate into T helper (Th) 1 or Th2 subsets with mucosal and systemic activity, but also in the Th17 and Th22 subsets that are mostly mucocutaneous. Secretion of interleukin (IL)-6 and TGFβ by dendritic cells induces both Th17 differentiation and IgA secretion, although the latter can also be induced by the more Th2-related IL-5 and IL-10 (Protti et al., 2014; Kiyono and Azegami, 2015). Finally, innate lymphoid cells play important roles in mucosal immunity and are found in high numbers in mucosal epithelia (Wu et al., 2014). These features of mucosal immunity have to be accounted for in the design of mucosal vaccines. While a dozen nasal (e.g., the Flumist^©^ anti-influenza spray) and enteric (rotavirus, poliovirus, cholera and salmonella) vaccines are already in clinical use (Lavelle and Ward, 2022; Pilapitiya et al., 2023), ongoing investigations explore buccal (defined here as targeting one or several subcompartments of the oral cavity), vaginal and rectal applications.

**Figure 1:**
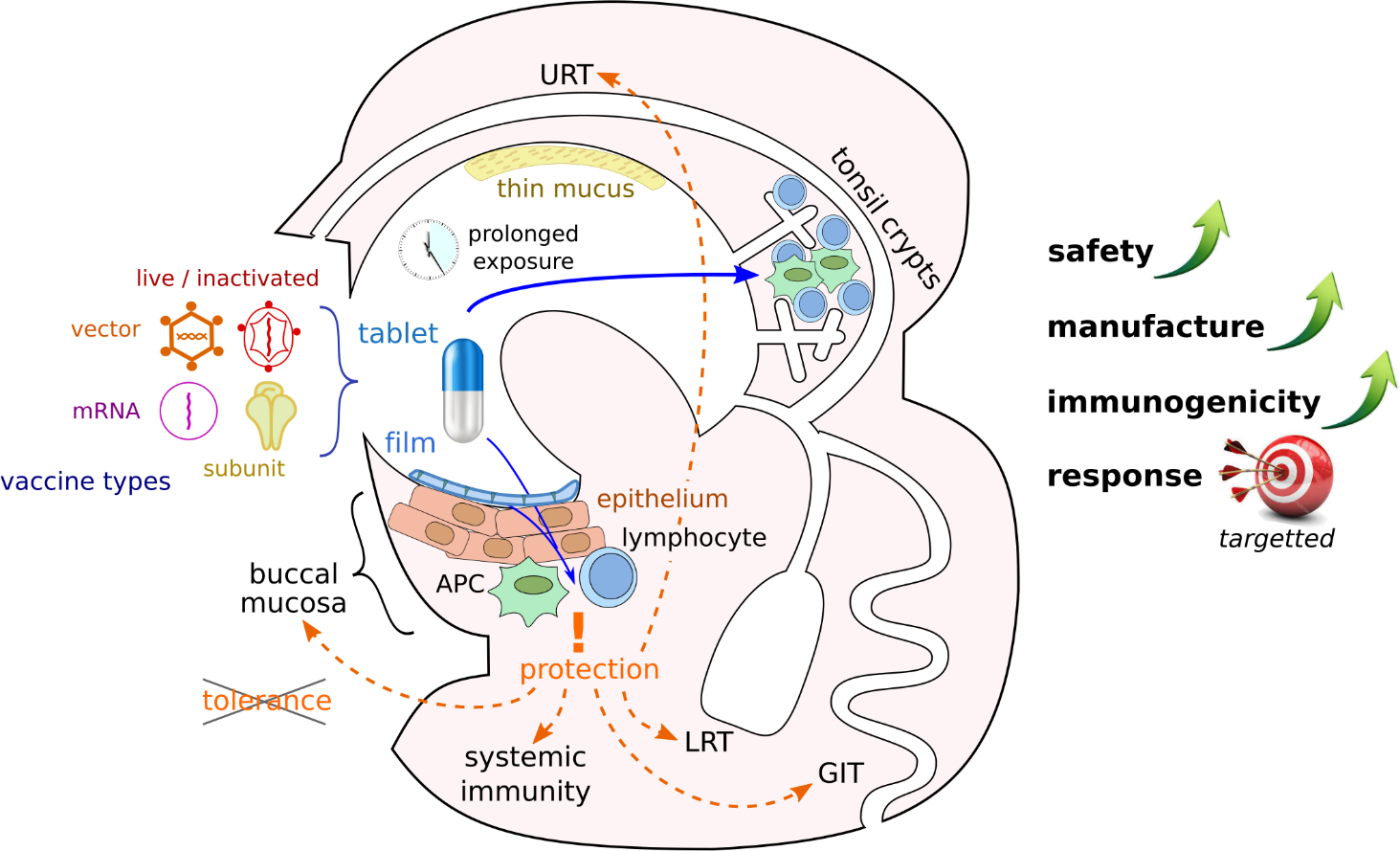
Advantages of buccal vaccination. Various vaccine types could be formulated as slowly dissolving pills or films and administered to the buccal mucosa. Crossing of mucus and epithelial barriers can facilitate antigen presentation to the immune system in different locations of the buccal cavity and lead to immune response in various organs if peripheral tolerance is avoided (APC, antigen presenting cell; URT, upper respiratory tract; LRT, lower respiratory tract; GIT, gastro-intestinal tract).

The sublingual route, in particular, offers critical advantages, including a reduced risk of anaphylactic shock and Bell’s palsy (Kweon, 2011); in humans, a non-keratinized epithelium facilitating rapid absorption of the vaccine (Paris et al., 2021); and a thin mucus layer that inactivates fewer antigens than in other mucosae (**Figure 1**). In addition, the buccal lymphoid tissue displays a unique ability of inducing not only local, but also systemic immunity and responses in most other mucosae (upper and lower respiratory, gastrointestinal and genital tracts) leading to multi-organs protections (Hervouet et al., 2010; Czerkinsky et al., 2011; Tsai et al., 2023). This is achieved through the selective migration of IgA-secreting plasmablasts, as well as antigen-carrying dendritic cells to secondary lymphatic organs [reviewed in (Paris et al., 2021)].

**Figure 2:**
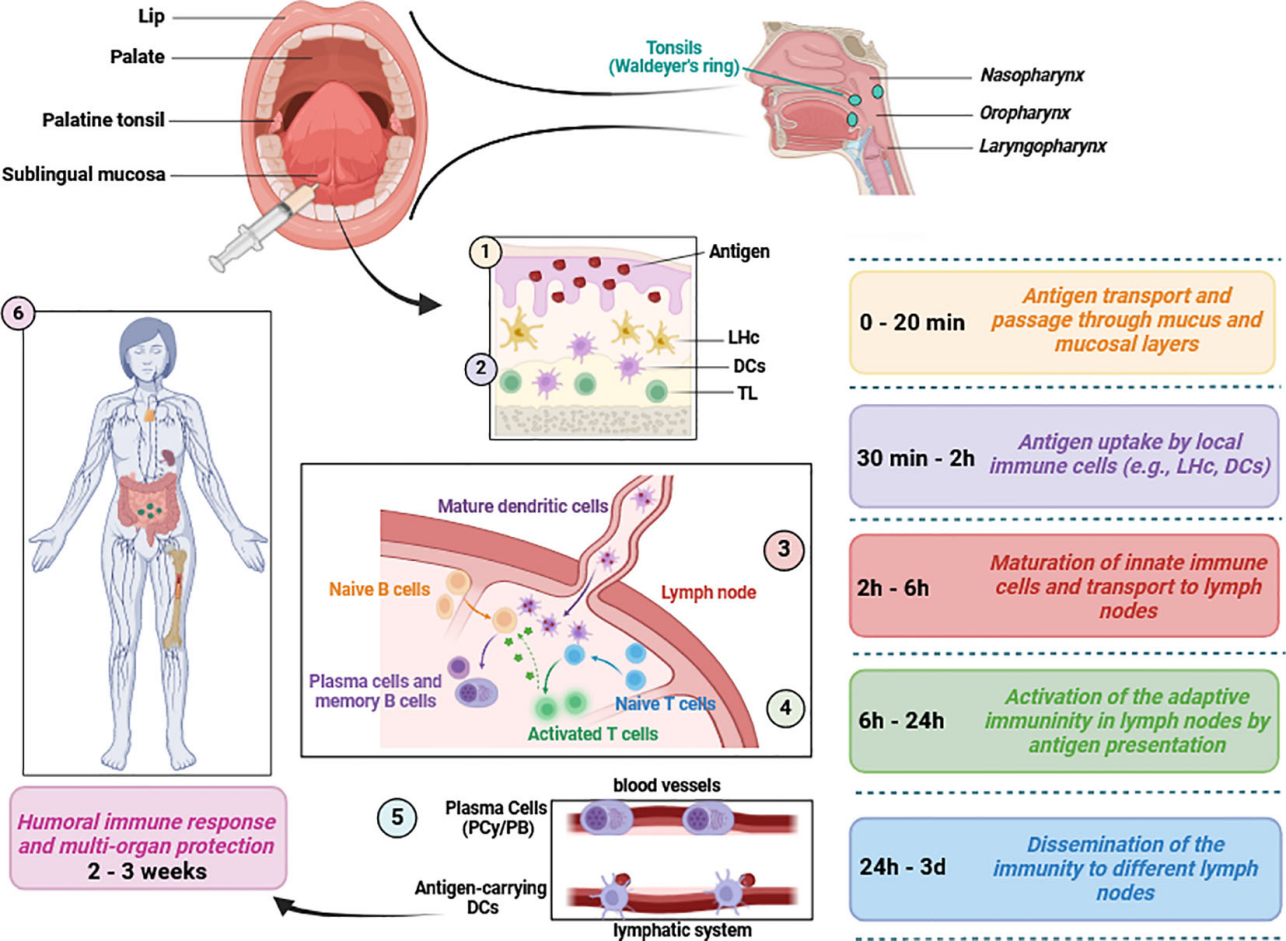
Mucosal immunity induction in response to sublingual vaccine application. Several mediators of both innate and adaptive immunity are involved following vaccine application. Typically, humoral defense mechanisms and immune protection occur within 2 to 3 weeks. This figure aims to briefly present the relevant immunological pathways and does not include detailed information on chemokines, cytokines or immune cell subsets (Paulsen and Waschke, 2010; Aydin et al., 2021; Paris et al., 2021; Baehren et al., 2022) LHc, Langerhans cells; DCs, dendritic cells including plasmacytoid, conventional and monocyte-derived subsets; PCy, Plasmacytes; PB, Plasmablasts; TL, T lymphocytes/cells. This figure was created using BioRender.com.

It is important to highlight that the sublingual method of vaccine application not only enables rapid vaccine absorption within 20 minutes but also represents an important step in overcoming the first metabolic passage (first-pass effect) (Paris et al., 2021). Within the first two hours post-application, a critical release of inflammatory cytokines and chemokines occurs, which play a crucial role in the maturation and activation of immune cells, including MHC-II^+^, CD11b^+^, and CD11c cells (Paris et al., 2021). The intensity of the immune response is largely dependent on the quantity of dendritic cells and the effectiveness of antigen uptake (Paris et al., 2021). The early immune response initiated by dendritic cells leads to the activation and proliferation of B and T lymphocytes (Paris et al., 2021). This results in the spread of antigen-specific effector cells to various lymph nodes (Paris et al., 2021). The humoral response typically manifests within three weeks following sublingual vaccination (Paris et al., 2021) (**Figure 2**). However, enteric and inhaled vaccines deliveries can also stimulate the buccal mucosa along their paths, even it is not their intended target. Sublingual vaccines should also simplify manufacture and deployment since no delivery equipment is needed and non-invasive, pain-free and needle-free administration reduces the workload for healthcare professionals and enhances public approval (Braun et al., 2023).

Sublingual delivery has long been the gold standard for allergen-specific immunotherapy in allergology departments and outpatient paractices, with several commercially available products targeting house dust mites and pollen. Briefly, these immunotherapies aim at redirecting the allergic IgE-dominant response towards IgA and IgG dominant, Th1-biased protective immunity following the preferential activation of regulatory dendritic cells by high dose allergen and subsequent induction of TGFβ- and IL10-expressing regulatory T cells (Durham and Shamji, 2023). Since these pathways partially match those required for efficient vaccination, despite their reliance on repeated administration and anti-inflammatory pathways that are not desired for vaccines, the knowledge on mucosal immunity and product formulation acquired with sublingual allergen immunotherapy may assist buccal vaccines development. In addition, the sublingual route is utilized to administer bacterial suspensions against wheezing (Nieto et al., 2021) or urinary tract bacterial infections (Lorenzo-Gomez et al., 2015), notably the approved Uromune^©^ treatment, that harness trained innate immunity to protect urinary or lower respiratory tract mucosae.

Despite these successes, only a limited number of clinical trials of buccal vaccines have been conducted to date. Two phase I trials (NCT02052934, NCT03548064) tested a subunit vaccine against *E.coli* but found the enteric route to be more immunogenic than sublingual delivery (Bernstein et al., 2019). However, in the NCT01488188 and NCT03017378 trials, sublingual and nasal deliveries of respectively the Flumist^©^ influenza live-attenuated vaccine and the TB/FLU-01L influenza-vectored tuberculosis vaccine proved comparably safe and immunogenic (Paris et al., 2021). Moreover, in the NCT01443936 trial, administration of an HAdV-E4 adenovirus vector encoding H5 influenza hemagglutinin to tonsils proved superior to the oral route in terms of antibodies and memory induction and equal to the intranasal one, even at a low vector dose (Matsuda et al., 2019, Matsuda et al., 2021). In our opinion, buccal delivery thus represents a priority target for which a next generation of vaccines should be optimized.

Addressing this goal will require extensive investigations across several topics to overcome the obstacles that have until now burdened buccal vaccines, including poor mucosa penetration, limited immunogenicity or impractical vaccine production.

First, the behavior of various vector types (e.g., adenovirus vectors) in the buccal environment and their interactions with mucosal resident immunity remain insufficiently understood in comparison to systemic delivery. Most sublingual vectorized vaccines tested so far have been constructed from standard backbones (notably HAdV-C5 and *B. subtilis*) with little effort of optimization for the specificities of mucosal environments including the risks of neutralization by antimicrobial peptides, mucus trapping and insufficient epithelium penetration (Kraan et al., 2014). For example, the AstraZeneca adenovirus-vectored COVID vaccine, although highly immunogenic intramuscularly, proved insufficiently immunogenic as an intranasal vaccine (Madhavan et al., 2022), while the Bharat intranasal COVID vaccine, derived from another adenovirus type, reached market approval and, although head-to-head comparisons are missing, displayed a higher efficacy than certain intramuscular vaccines (Singh et al., 2023). Thus, vector engineering is a largely untapped but promising area of investigations. Research on buccal vaccines has predominantly focused so far on subunit vaccines paired with adjuvants, such as *V. cholerae* and *E.coli* toxin derivatives, whose safety remains nevertheless controversial (Mutsch et al., 2004). Other adjuvants including chitosan (Mohamed et al., 2018; Gong et al., 2022), which helps antigens to cross the mucus layer, and flagellin (Khim et al., 2023), a potent stimulant of mucosal innate immunity, are thus increasingly used. Optimized antigens and adjuvants may in the future be incorporated into more complex platforms like adenoviral vectors to synergistically enhance their advantages. Substantial advances have been made in the last decade in viral and bacterial vectors engineering (Capasso et al., 2016; Guiziou et al., 2016; Wang et al., 2019), antigen design (Cohen et al., 2022), and immunostimulatory peptide knowledge (Weiss et al., 2022), already showing success in synthetic biology, gene therapy or cancer treatment, and the application of these methods to mucosal vaccination may bring about a new wave of success.

Second, it is important to identify and avoid conditions in which antigen presentation leads to peripheral tolerance (Tsai et al., 2023). Interestingly, the sublingual mucosa appears to be less prone to peripheral tolerance than the intestine (Tsai et al., 2023). Furthermore, virus- to bacteria-sized particles appear less tolerogenic than isolated proteins (Paris et al., 2021), indicating that buccal vaccine research may benefit from focusing on vector vaccines. Ideally, these vectors would be derived from microorganisms adapted to the target mucosal environment, since their coevolution with the immune system can be expected to render them immunogenic and efficient at transducing the mucosa.

Third, sublingual vaccine components need to remain in contact with buccal mucosa for several minutes to efficiently transduce it. The failure of the buccal route in several vaccine trials may be attributed (alongside the use of vaccines originally designed for other routes) to the use of liquid formulation, leading to the quick dispersal of vaccine components (Huo et al., 2012; Bernstein et al., 2019). Furthermore, the buccal environment presents staggering individual differences due to the influence of nutrition, drinking, smoking etc. on saliva chemical composition as well as to the abundant microbiota and related possibilities of lesions and inflammation. All these factors may interfere with vaccine compounds release and absorption and thus lead to variable biodistribution with deleterious consequences on immunization success rate. Vaccine formulation should therefore include biodegradable mucoadhesive films, patches, microneedles or tablets that help buffer against the variability of mucosal environments and have already facilitated substantial improvements in exposure elongation (Huo et al., 2012; Jacob et al., 2021). The next high-impact goals in formulation are now production upscaling and adaptation to high-molecular weights vaccine types (vectors, mRNA, DNA) whose incorporation at high concentration in the delivery system and intact mucosal release are still challenging.

Fourth, representative models of the human mucosae are urgently needed. The oral cavity, which is covered by mucosal epithelium, extends from the lips to the tongue and circumvallate papillae on the tongue, oral flora, buccal mucosa, retromolar space, and includes the hard and soft palates, as well as mylohyoid muscle and buccomasseter (Kiyono and Azegami, 2015; Montero and Patel, 2015; Famuyide et al., 2022). This cavity also functions as a secondary airway and provides continuous defense against pathogens, supported by the mucosal immune system (Kiyono and Azegami, 2015; Famuyide et al., 2022). The pharynx is divided into nasopharynx, oropharynx, and laryngopharynx (Morris, 1988; Baehren et al., 2022). The tonsils, which are located on the isthmus faucium, at the lateral walls and at the back of the tongue and play an important role in immune defense (Morris, 1988; Fossum et al., 2017; Aydin et al., 2021; Baehren et al., 2022).

Mice models have been proven to poorly predict the consequences of sublingual and intranasal vaccination in humans (Kraan et al., 2014; Jameson and Masopust, 2018) due to substantial differences in immune response (Cai et al., 2022) and in respiratory and digestive tracts anatomy (Tuero and Robert-Guroff, 2014). Non-human primates (NHPs), although used for certain preclinical tests, present ethical challenges and were generally less predictive of mucosal vaccines results in human than they have been for systemic vaccination (Kayesh et al., 2021). The tree shrew *Tupaia belangeri*, already used to study numerous human pathogens (Kulkarni et al., 2009), may represent an acceptable compromise, being anatomically and phylogenetically closer to humans than rodents while easing the logistical and ethical burden of NHPs. Similarly, pigs are considered representative models of human buccal anatomy (Wagar et al., 2021). However, immunological studies in these models are impractical since dedicated reagents are rare.

The current lack of satisfactory animal models underscores the interest of human *ex vivo* models that can model native mucosal immune response and even inter-individual variability. In particular, cultures of primary lymphoid tissues from tonsillectomies have already been established and harnessed (Perry, 1994; Kastenschmidt et al., 2023). Tonsillar crypts, with their large contact surface at the crossroads between respiratory and digestive tracts and a reticulated epithelium intertwined with immune cells infiltrates (Perry, 1994), indeed represent a primary target for immunization. Efficient delivery methods such as mucoadhesive patches and microneedles are equally applicable to the sublingual compartment as to tonsils, making the latter a valid clinical target. Furthermore, the easily obtained and cultured tonsil lymphoid organoids may be associated with epithelia representative of native crypt architecture to obtain models that could facilitate the mechanistic understanding of mucosal immunization and the optimization of new vaccines. Owing to the extensive interconnexions between mucosal compartments, tonsil lymphoid organoids could alternatively be cultivated with sublingual or even non-buccal mucosal epithelium to better model the intended vaccine delivery site.

Fifth, the mucosal effector and memory response to vaccines needs to be deciphered in more detail. For instance, the involvement of dendritic and M cells in vaccine antigen uptake and presentation has been well-described in the intestine (Islam et al., 2019; Lavelle and Ward, 2022), but knowledge from other mucosae lags behind.

To conclude, there is a growing need for non-canonical delivery routes. Breakthroughs have occurred during the last years in nasal and oral vaccination, building on progress in immunology and the scientific and industrial experience acquired during the COVID pandemic, and similar advances may be achieved in a foreseeable future to harness the unique opportunities of the buccal route. In particular, targeted high-throughput vector engineering, adjuvant optimization and mucoadhesive formulations appear to be promising research fields that may contribute to make mucosal vaccination a potent tool in combatting current diseases, the antimicrobials resistance crisis, and future emerging diseases. Integrated approaches considering pharmacology, immunology and manufacture constraints already at early stages are warranted to unleash the full potential of next-generation vaccinology.

## Author contributions

MA: Writing – review & editing, Writing – original draft, Validation, Supervision, Project administration, Conceptualization. ES: Writing – review & editing, Writing – original draft, Visualization, Conceptualization.
